# Prevalence, incidence, and outcomes of hepatitis E virus coinfection in patients with chronic hepatitis C

**DOI:** 10.1038/s41598-023-39019-3

**Published:** 2023-08-21

**Authors:** Eun Sun Jang, Gwang Hyeon Choi, Young Seok Kim, In Hee Kim, Youn Jae Lee, Sung Beom Cho, Yun-Tae Kim, Sook-Hyang Jeong

**Affiliations:** 1 Department of Internal Medicine, Seoul National University College of Medicine, Seoul National University Bundang Hospital, Seongnam, Gyeonggi Republic of Korea; 2 Department of Internal Medicine, Soonchunhyang University Bucheon Hospital, Soonchunhyang University College of Medicine, Bucheon, Gyeonggi Republic of Korea; 3 Department of Internal Medicine, Chonbuk National University Hospital, Chonbuk National University College of Medicine, Chonju, Republic of Korea; 4 Department of Internal Medicine, Inje University Busan Paik Hospital, Inje University College of Medicine, Busan, Republic of Korea; 5 Department of Internal Medicine, Chonnam National University Hwasun Hospital, Jeonnam, Republic of Korea; 6 Center for Technology Innovation, Seoul Clinical Laboratories, Yongin, Gyeonggi Republic of Korea

**Keywords:** Hepatology, Hepatitis C virus, Viral epidemiology

## Abstract

This study aimed to elucidate the anti-hepatitis E virus (HEV) immunoglobulin G (IgG) prevalence and incidence of seroconversion and seroreversion as well as its risk factors and to analyze the clinical outcomes of HEV and hepatitis C virus (HCV) coinfected patients compared to those of HCV-monoinfected patients. We prospectively enrolled 502 viremic HCV patients with paired plasma samples (at intervals of ≥ 12 months) from 5 tertiary hospitals. Anti-HEV IgG positivity was tested using the Wantai ELISA kit in all paired samples. Mean age was 58.2 ± 11.5 years old, 48.2% were male, 29.9% of patients had liver cirrhosis, and 9.4% of patients were diagnosed with hepatocellular carcinoma (HCC). The overall prevalence of anti-HEV IgG positivity at enrollment was 33.3%, with a higher prevalence in males and increasing prevalence according to the subject’s age. During the 916.4 person-year, the HEV incidence rate was 0.98/100 person-years (9/335, 2.7%). Hepatic decompensation or liver-related mortality was not observed. There were six seroreversion cases among 172 anti-HEV-positive patients (1.22/100 person-years). In conclusion, approximately one-third of the adult Korean chronic HCV patients were anti-HEV IgG positive. The HEV incidence rate was 1 in 100 persons per year, without adverse hepatic outcomes or mortality.

## Introduction

Hepatitis E virus (HEV) is an important cause of acute viral hepatitis that occurs through fecal-to-oral transmission^1,2^. Globally, there are approximately 20 million cases of HEV infection every year, with 3.3 million symptomatic cases (less than 20%) which resulted in more than 40,000 deaths in 2015^3^. The age-standardized global incidence rate of HEV infection in 2019 was 271.2 and 263.4 per 100,000 person-years for males and females, respectively, with considerable geographic variation^4^.

Although the overall case-fatality of HEV infection is low except for pregnant women, it has been reported that superimposed HEV infection resulted in higher mortality of underlying chronic hepatitis B patients in Hong Kong^5^ and Taiwan^6^. Interestingly, HEV coinfection did not increase the mortality of hepatitis C patients in the same study^5^ and did not contribute to hepatic decompensation in advanced chronic hepatitis C patients in the US^7^.

The anti-HEV prevalence in the South Korean general population aged 10–55 years was 5.9% during 2007–2009, showing increasing prevalence by age: 1.2% in the 20s, 2.4% in the 30s, 12.0% in the 40s, and 20.9% in the 50s^8^. However, there are no data on the prevalence and incidence of superimposed HEV infection among chronic hepatitis C patients in South Korea. Moreover, whether HEV coinfection affects the outcomes of HCV infection is not known. Therefore, this study aimed to elucidate anti-HEV IgG prevalence and its related factors, to assess the anti-HEV IgG seroconversion and reversion incidence rates and to analyze outcomes of HEV and HCV coinfected patients compared to those of HCV monoinfected patients.

## Results

### Baseline characteristics of the HCV-infected patients

The mean age of the 502 subjects was 58.2 ± 11.5 years old, and 242 (48.2%) were males. At the time of initial HEV testing, liver cirrhosis and HCC were found in 29.9% and 9.4% of patients, respectively (Table 1).

**Table 1:** Baseline characteristics of the 502 chronic hepatitis C-infected patients.

Characteristics	Total (n = 502)	Anti-HEV IgG (−) (n = 335)	Anti-HEV IgG (+) (n = 167)	P
Age	58.2 ± 11.5	55.2 ± 11.4	64.4 ± 9.0	** < 0.001**
Male	242 (48.2)	145 (43.3)	97 (58.1)	**0.002**
Liver diseases				**0.001**
Chronic hepatitis	305 (60.8)	222 (66.3)	83 (49.7)	
Compensated cirrhosis	139 (27.7)	85 (25.4)	54 (32.3)	
Decompensated cirrhosis	11 (2.2)	5 (1.5)	6 (3.6)	
Hepatocellular carcinoma	47 (9.4)	23 (6.9)	24 (14.4)	
APRI	0.68 (0.37–1.33)	0.58 (0.35–1.31)	0.74 (0.44–1.44)	0.104
≥ 0.5	308 (61.4)	193 (58.2)	115 (68.9)	**0.045**
FIB-4	2.56 (1.58–4.64)	2.22 (1.32–4.39)	3.08 (2.07–5.16)	**0.001**
≥ 1.45	199 (39.6)	119 (35.5)	80 (47.9)	** < 0.001**
HCV genotype				0.084
1/2	253 (50.4)/229 (45.6)	175 (52.2)/148 (44.2)	78 (46.7)/81 (48.5)	
3/4	2 (0.4)/1 (0.2)	2 (0.6)/1 (0.3)	0 (0)/0 (0)	
6/mixed	4 (0.8)/1 (0.2)	4 (1.2)/1 (0.3)	0 (0)/0 (0)	
Missing	12 (2.4)	4 (1.2)	8 (4.8)	
HCV RNA, Log_10_ IU/mL	5.69 (4.29–6.39), n = 496	5.67 (4.30–6.40), n = 332	5.64 (4.24–6.32), n = 164	0.557
Treatment history				0.248
No	369 (73.5)	238 (71.0)	131 (78.4)	
Interferon-based, no SVR	65 (12.9)	49 (14.6)	16 (9.6)	
Interferon-based, SVR	49 (9.8)	36 (10.7)	13 (7.8)	
DAA, SVR	19 (3.8)	12 (3.6)	7 (4.2)	
Diabetes mellitus	84 (16.7)	52 (15.5)	32 (19.2)	0.303
Body mass index, kg/m^2^	23.9 ± 3.4, n = 484	23.7 ± 3.3, n = 323	24.3 ± 3.5, n = 159	0.095
Obesity (≥ 25.0 kg/m^2^)	159 (32.9)	95 (29.4)	64 (39.8)	**0.022**
Alcohol				0.364
No	244 (48.6)	160 (47.8)	84 (50.3)	
Social	159 (31.7)	103 (30.7)	56 (33.5)	
Significant	99 (19.7)	72 (21.5)	27 (16.2)	
Ever smoking	218 (43.4)	143 (42.7)	75 (44.9)	0.636
Laboratory findings				
WBC, ×1000/mm^3^	5.2 (4.2–6.5)	5.2 (4.3–6.5)	5.1 (4.1–6.5)	0.416
Hemoglobin, g/dL	13.7 (12.6–14.7)	13.7 (12.7–14.8)	13.6 (12.3–14.6)	0.068
Platelet, ×1000/mm^3^	168 (127—211)	176 (133–219)	152 (116–199)	**0.001**
Albumin, g/dL	4.2 (4.0–4.5)	4.3 (4.0–4.5)	4.1 (3.8–4.4)	**0.014**
Bilirubin, mg/dL	0.8 (0.6–1.0)	0.7 (0.6–1.0)	0.8 (0.6–1.0)	0.600
ALP, IU/L	90 (71–135)	91 (71–141)	90 (72–124)	0.095
AST, IU/L	43 (29–69)	42 (28–67)	45 (31–73)	0.922
ALT, IU/L	35 (22–58)	35 (23–58)	33 (21–59)	0.484
γ-gt, IU/L	41 (22–81), n = 401	41 (20–90), n = 264	40 (24–71), n = 137	0.167
Prothrombin time, INR	1.06 (1.00–1.12), n = 492	1.05 (0.99–1.12), n = 327	1.07 (1.02–1.14), n = 165	**0.010**
Creatinine, mg/dL	0.82 (0.70–1.00)	0.80 (0.67–0.99)	0.90 (0.70–1.00)	0.449
Alpha-fetoprotein, ng/dL	3.7 (2.4–8.6), n = 492	3.7 (2.4–8.1), n = 328	3.6 (2.2–11.2), n = 164	0.507
Antiviral treatment^a^	357 (71.1)	241 (71.9)	116 (69.5)	0.819
Interferon-based, no SVR	34 (6.8)	26 (7.8)	8 (4.8)	
Interferon-based, SVR	37 (7.4)	24 (7.2)	13 (7.8)	
DAA, no SVR	20 (4.0)	13 (3.9)	7 (4.2)	
DAA, SVR	293 (58.4)	198 (59.1)	95 (56.9)	

HEV, hepatitis E virus; APRI, aspartate aminotransferase platelet ratio index; FIB-4, fibrosis-4 index; HCV, hepatitis C virus; RNA, ribonucleic acid; SVR, sustained virologic response; DAA, direct acting antivirals; WBC, white blood cell; ALP, alkaline phosphatase; AST, aspartate aminotransferase; ALT, alanine aminotransferase; γ-gt, gamma-glutamyl transferase; INR, international normalized ratio.

Significant values are in bold.

^a^Twenty-seven patients received both interferon-based treatment and subsequent DAA treatment during follow-up period.

The median HCV RNA level was 5.69 log_10_ IU/mL, and 96% of the subjects were infected with HCV genotype 1 or 2. A significant number of alcohol drinkers accounted for 19.7% of the study population, and 43.4% of the patients had a history of smoking. Before and after enrollment, 71.1% of the patients had received antiviral therapy. Among the patients that had received antiviral therapy, 14.2% of the patients were treated with an interferon-based regimen, 62.4% of the patients were treated with direct-acting antivirals (DAA), and 5.4% of the patients were treated with both regimens. The overall SVR rate was 92.4% during the follow-up period.

### Prevalence and predictive factors of anti-HEV IgG positivity in chronic HCV-infected patients

**Figure 1:**
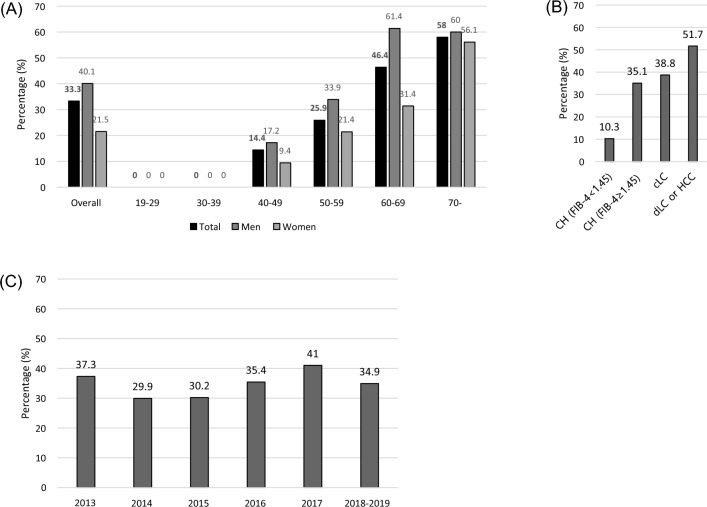
Prevalence of anti-HEV immunoglobulin G in Korean patients with chronic hepatitis C virus infection according to age group and sex (**A**), underlying liver disease status (**B**), and the year of the blood sample collected (**C**). (**A**) Black, overall; Dark gray, men; Light gray, women, (**B**) CH, chronic hepatitis; cLC, compensated liver cirrhosis; dLC, decompensated liver cirrhosis; HCC, hepatocellular carcinoma, FIB-4; fibrosis-4 index.

At the time of enrollment, the overall prevalence of positive anti-HEV IgG was 33.3% (167/502). It increased according to subjects’ age: 0% for those ≤ 30, 14.4% for those in their 40s, 25.9% for those in their 50s, 46.4% for those in their 60s, and 58% for those ≥ 70 (p < 0.001, Table 1). As shown in Fig. 1, the anti-HEV IgG prevalence was higher in men for all age groups (p = 0.001, Fig. 1, Table S1).

We collected detailed information about employment and analyzed whether a specific occupation was related to the high HEV prevalence. Administrative workers, farmers, and fishermen were more likely to present with anti-HEV IgG positivity, but individuals in those occupations were significantly older than those in other occupations (data not shown). The prevalence of obesity (≥ 25.0 kg/m^2^) was significantly higher in the anti-HEV-positive group than in the anti-HEV-negative group (39.8% vs. 29.4%, p = 0.022). The prevalence of advanced liver diseases, including liver cirrhosis and HCC, was higher in the anti-HEV-positive group (p = 0.001).

Of the laboratory findings, platelet (152 vs. 176 ×1000/mm^3^, p = 0.001) and albumin (4.1 vs. 4.3 g/dL, p = 0.014) levels were lower, and the prothrombin time international normalized ratio (PT INR) was higher (1.07 vs. 1.05, p = 0.01) in the anti-HEV-positive group than in the negative group. Similarly, patients with a high APRI (≥ 0.5) and a high FIB-4 score (≥ 1.45) were more common in the anti-HEV IgG-positive group than in the negative group (Table 1). HCV genotype and viral load were similar in both groups.

According to the multivariable logistic regression analysis, older age (odds ratio [OR] 1.10, 95% 1.07–1.12), male sex (OR 2.06, 95% CI 1.34–3.16), and obesity (OR 1.89, 95% CI 1.21–2.95) were independent factors for anti-HEV IgG positivity in patients with chronic HCV infection (Table 2). Furthermore, these three variables were consistently significant factors for anti-HEV IgG positivity in the other three multivariable models. However, liver cirrhosis or serologic liver fibrosis markers (FIB-4 or APRI) were not independent factors for anti-HEV IgG positivity.

**Table 2:** Independent predictors of positive anti-HEV IgG in patients with chronic hepatitis C infection.

Variables	Univariate analysis		Multivariate model 1		Multivariate model 2		Multivariate model 3		Multivariate model 4	
	Odd ratio (95% CI)	P	Odd ratio (95% CI)	P	Odd ratio (95% CI)	P	Odd ratio (95% CI)	P	Odd ratio (95% CI)	P
Age	1.09 (1.07–1.11)	< 0.001	1.10 (1.07–1.12)	< 0.001	1.10 (1.07–1.12)	< 0.001	1.09 (1.07–1.12)	< 0.001	1.10 (1.07–1.12)	< 0.001
Men	1.82 (1.25–2.64)	0.002	2.06 (1.34–3.16)	0.001	2.05 (1.33–3.16)	0.001	2.10 (1.37–3.22)	0.001	2.13 (1.39–3.27)	0.001
Liver cirrhosis	1.99 (1.36–2.90)	< 0.001	1.46 (0.95–2.24)	0.083	1.24 (0.74–2.06)	0.418	–	–	–	–
FIB-4 ≥ 1.45	4.96 (2.63–9.36)	< 0.001	–	–	–	–	1.56 (0.75–3.24)	0.231	–	–
APRI ≥ 0.5	1.63 (1.10–2.41)	0.015	–	–	–	–	–	–	1.19 (0.75–1.91)	0.463
Obesity	1.58 (1.07–2.35)	0.023	1.89 (1.21–2.95)	0.005	1.93 (1.23–3.03)	0.004	1.87 (1.19–2.92)	0.006	1.87 (1.20–2.92)	0.006
Platelet	0.995 (0.992–0.998)	0.001	–	–	0.997 (0.993–1.001)	0.171	–	–	–	–
ALT	1.000 (0.997–1.003)	0.922	–	–	–	–	–	–	–	–
Albumin	0.57 (0.38–0.90)	0.015	–	–	1.11 (0.64–1.92)	0.721	0.94 (0.56–1.58)	0.809	0.94 (0.55–1.62)	0.823
Total bilirubin	0.91 (0.65–1.28)	0.603	–	–	–	–	–	–	–	–

HEV, hepatitis E virus; IgG, immunoglobulin G; CI, confidence interval; FIB-4, fibrosis-4 index; APRI, aspartate aminotransferase platelet ratio index; ALT, alanine aminotransferase.

### Incidence of anti-HEV IgG seroconversion and reversion rate in chronic HCV-infected patients

During the median 2.4 (IQR 1.7–3.8) years of the interval-time period, 9 cases (2.7%) showed seroconversion from anti-HEV negativity to positivity. The incidence rate of new HEV infection was 0.98 (95% CI 0.51–1.88)/100 person-years: 0.91, 0.95, 1.33, and 1.21/100 person-years in patients in their 40s, 50s, 60s and 70s, respectively (Table 3).

**Table 3:** Incidence of anti-HEV IgG seroconversion and seroreversion in patients with chronic hepatitis C infection.

Variables	Rate, per 100 person-years (95% CI)	Anti-HEV IgG sero-conversion or reversion (cases)	Person-year	P-value
Seroconversion (from negative to positive)				
Total	0.98 (0.51–1.88)	9	916.4	
Sex				0.667
Women	0.73 (0.28– 1.95)	4	545.8	
Men	1.35 (0.56–3.22)	5	370.6	
Age group				0.317
19–29	0	0	26.2	
30–39	0	0	45.0	
40–49	0.91 (0.23–3.61)	2	220.3	
50–59	0.95 (0.31–2.93)	3	316.3	
60–69	1.33 (0.43–4.09)	3	225.9	
70-	1.21 (0.17–8.49)	1	82.6	
Seroreversion (from positive to negative)				
Total	1.22 (0.55–1.88)	6	492.7	
Sex				0.741
Women	1.40 (0.45–4.30)	3	214.4	
Men	1.08 (0.35–3.32)	3	278.3	
Age group				0.998
40–49	2.46 (0.36–17.07)	1	40.6	
50–59	1.54 (0.39–6.10)	2	129.8	
60–69	0.47 (0.07–3.35)	1	211.2	
70-	1.80 (0.46–7.10)	2	111.1	

HEV, hepatitis E virus; IgG, immunoglobulin; CI, confidence interval.

Detailed information about the nine seroconverted cases is described individually in Table 4. These patients had no HCC development or mortality during the entire follow-up period.

**Table 4:** Detailed characteristics of 9 anti-HEV IgG sero-converted cases in patients with chronic hepatitis C infection.

No	Baseline (anti-HEV IgG negative)											At seroconversion time^a^ (anti-HEV IgG positive)								
	Age	Sex	Job	HCV RNA (IU/mL)	PLT (×1000/mm^3^)	ALT (IU/L)	Alb (g/dL)	FIB-4	APRI	LC	HCC	Interval (year)	HEV IgG titer	HCV RNA (IU/mL)	PLT (×1000/mm^3^)	ALT (IU/L)	Alb (g/dL)	FIB-4	APRI	HCC
1	75	M	Technical	22,632	200	36	4.7	2.21	0.44	Yes	Yes	2.04	1.08	3310	144	54	4.4	2.72	0.64	Yes
2	61	F	Administrative	4,120,000	224	112	3.7	0.93	0.25	No	No	5.22	1.72	0	164	72	4.2	2.41	0.76	No
3	59	F	Housewife	0	94	26	3.6	4.00	0.85	Yes	No	4.88	13.31	0	103	25	4.5	3.53	0.68	No
4	57	M	N/A	2,257,000	206	39	4.2	5.05	2.21	Yes	No	2.71	1.13	1,258,746	169	51	3.9	3.52	1.04	No
5	46	M	N/A	271,000	234	398	5.0	1.26	1.34	No	No	5.32	5.16	0	259	21	4.8	0.92	0.20	No
6	48	F	Service	346,000	53	63	4.4	2.92	1.03	Yes	No	3.71	10.39	0	94	54	4.0	4.42	1.01	No
7	66	F	Housewife	2190	122	20	3.0	5.74	1.80	Yes	No	1.12	1.67	0	127	24	3.1	4.59	0.73	No
8	54	M	Administrative	111,588	158	49	4.5	3.17	0.97	No	No	4.21	1.60	0	143	8	4.5	3.20	0.38	No
9	61	F	Housewife	0	173	45	5.1	1.63	0.26	No	No	2.73	5.44	0	180	21	4.5	2.04	0.36	No

HEV, hepatitis E virus; IgG, immunoglobulin G; M, men; F, women; HCV, hepatitis C virus; RNA, ribonucleic acid; PLT, platelet; ALT, alanine aminotransferase; Alb, albumin; FIB-4, fibrosis-4 index; APRI, aspartate aminotransferase platelet ratio index; LC, liver cirrhosis; HCC, hepatocellular carcinoma; N/A, not available.

^a^There was no mortality neither new HCC development among 9 patients with anti-HEV IgG incidence case.

In addition, there were no significant differences in platelet count, ALT, FIB-4, and APRI after HEV IgG seroconversion (Fig. S1).

Anti-HEV IgG seroreversion was observed in 6 subjects with incidence rates of 1.22 (0.55–1.88)/100 person-years: 2.46, 1.54, 0.47, and 1.80 per 100 person-years in patients in their 40s, 50s, 60s and 70s, respectively (Table 3). As shown in Table 5, 4 of the 6 seroreverted patients with a low IgG titer (< 2) initially showed negative results at the follow-up test, suggesting a remote infection or a possible false positivity at baseline. The remaining two seroreverted cases were obese males in their 50s. No remarkable characteristics differed from those without seroreversion regarding their occupation, HCV RNA levels, liver cirrhosis, or fibrosis markers (Table 5).

**Table 5:** Detailed characteristics of 6 anti-HEV IgG sero-reverted cases in patients with chronic hepatitis C infection.

No	Baseline (anti-HEV IgG positive)												After seroreversion (anti-HEV IgG negative)							
	Age	Sex	Job	HCV RNA (IU/mL)	PLT (×1000/mm^3^)	ALT (IU/L)	Alb (g/dL)	FIB-4	APRI	LC	HCC	HEV IgG titer	Interval(yr)	HCV RNA (IU/mL)	PLT (×1000/mm^3^)	ALT (IU/L)	Alb (g/dL)	FIB-4	APRI	HCC
1	80	F	Housewife	1,334,087	74	82	3.6	11.02	3.11	Yes	No	1.13	1.82	577,344	60	44	3.3	9.49	1.92	No
2	69	F	Housewife	3,357,985	264	17	3.7	3.13	0.46	No	No	1.54	5.56	7,660,000	209	27	4.2	6.63	1.15	No
3	51	M	Service	193,663	176	115	4.1	2.07	1.07	No	No	4.54	2.31	0	161	77	4.6	1.69	0.68	No
4	78	M	None	0	216	16	4.7	2.35	0.30	No	No	1.18	1.44	0	268	34	4.0	1.53	0.28	No
5	45	F	Housewife	3,060,000	34	19	3.1	19.85	4.71	Yes	No	1.42	2.31	1,600,000	32	15	3.3	19.87	3.98	No
6	51	M	Industry worker	3,372,807	173	193	4.1	3.54	2.38	Yes	No	14.79	2.05	0	213	28	4.6	1.62	0.40	No

HEV, hepatitis E virus; IgG, immunoglobulin G; M, men; F, women; HCV, hepatitis C virus; RNA, ribonucleic acid; PLT, platelet; ALT, alanine aminotransferase; Alb, albumin; FIB-4, fibrosis-4 index; APRI, aspartate aminotransferase platelet ratio index; LC, liver cirrhosis; HCC, hepatocellular carcinoma.

### Comparison of the outcomes between the anti-HEV IgG positive and negative groups

Outcome assessments were performed in 444 patients (299 anti-HEV IgG negative and 145 anti-HEV IgG positive), excluding those with HCC or decompensated cirrhosis at baseline. During the 5.4 (3.2–6.8) year follow-up period, 30 patients developed HCC (6.8%), 6 patients developed decompensation (1.4%), and 17 died (3.8%; 11 liver-related and 6 non-liver related deaths). The annual incidence rates of HCC development, hepatic decompensation, and all-cause mortality were 1.39 (0.98–1.99), 0.27 (0.12–0.60), and 0.76 (0.47–1.22) per 100 person-years, respectively. There was no significant difference between the anti-HEV positive and negative groups in terms of HCC development (HR 1.74, 95% CI 0.84–3.58), hepatic decompensation (HR 2.22, 95% CI 0.45–10.98) or all-cause mortality (HR 0.68, 95% CI 0.22–2.07) (Fig. S2).

Because anti-HEV IgG positivity was related to the subject’s age, which is an essential predictor of HCC and mortality, we performed time-varying Cox analyses and propensity-score matching. Age, HCV genotype 1, a higher APRI (in model 1), or a lower platelet count (in model 2) were independent predictors of HCC and composite endpoints (including HCC development, hepatic decompensation, and mortality) according to our time-varying Cox analysis. However, anti-HEV IgG positivity did not predict these outcomes independently (Table 6).

**Table 6:** Time-varying Cox regression analysis for HCC and all-cause mortality in patients with chronic hepatitis C infection.

Variable	HCC^a^ (model 1)		Composite endpoint^b^ (model 1)	
	aHR (95% CI)	*P*	aHR (95% CI)	*P*
Anti-HEV IgG				
Negative	Reference	–	Reference	–
Positive	1.20 (0.56–2.57)	0.637	1.00 (0.54–1.88)	0.990
Age, year	1.06 (1.02–1.11)	**0.005**	1.05 (1.02–1.08)	**0.004**
Men	2.28 (0.72–7.20)	0.161	1.88 (0.80–4.44)	0.148
HCV genotype				
2	Reference	–	Reference	–
1	3.07 (1.22–7.73)	**0.017**	2.11 (1.08–4.12)	**0.029**
Achievement of SVR	0.49 (0.22–1.05)	0.066	0.49 (0.27–0.91)	**0.023**
APRI				
< 0.5	Reference	–	Reference	–
0.5–1.5	11.68 (1.53–89.29)	**0.018**	6.63 (1.99–22.11)	**0.002**
≥ 1.5	19.54 (2.53–150.95)	**0.004**	10.73 (3.13–36.76)	** < 0.001**
Ever smoker	1.61 (0.57–4.56)	0.374	1.81 (0.79–4.15)	0.159
Alcohol consumption				
None	Reference	–	–	–
Social	1.96 (0.66–5.75)	0.223		
Significant	1.48 (0.36–6.10)	0.589		
Albumin, g/dL	0.44 (0.17–1.11)	0.082	0.38 (0.18–0.79)	**0.009**

Variable	HCC^a^ (model 2)		Composite endpoint^b^ (model 2)	
	aHR (95% CI)	*P*	aHR (95% CI)	*P*
Anti-HEV IgG				
Negative	Reference	–	Reference	–
Positive	1.18 (0.55–2.56)	0.667	1.03 (0.55–1.92)	0.938
Age, year	1.07 (1.02–1.12)	**0.003**	1.05 (1.02–1.08)	**0.003**
Male	2.72 (0.73–7.11)	0.159	1.82 (0.77–4.30)	0.175
HCV genotype				
2	Reference	–	Reference	–
1	2.85 (1.14–7.10)	**0.024**	2.02 (1.03–3.95)	**0.040**
Achievement of SVR	0.48 (0.23–1.04)	0.062	0.49 (0.26–0.89)	**0.020**
Cirrhosis	0.95 (0.38–2.37)	0.904	1.04 (0.50–2.15)	0.923
Ever smoker	1.57 (0.55–4.49)	0.399	2.07 (0.91–4.70)	0.084
Alcohol consumption				
None	Reference	–	–	–
Social	2.31 (0.81–6.58)	0.117		
Significant	2.12 (0.51–8.77)	0.301		
Platelet, ×1000/mm^3^	0.991 (0.984–0.999)	**0.021**	0.991 (0.985–0.997)	**0.003**
Albumin, g/dL	0.43 (0.17–1.08)	0.074	0.40 (0.19–0.82)	**0.013**

HCC, hepatocellular carcinoma; aHR, adjusted hazard ratio; CI, confidence interval; HEV, hepatitis E virus; IgG, immunoglobulin G; SVR, sustained virologic response; APRI, aspartate aminotransferase platelet ratio index.

Significant values are in bold.

^a^Total number of patients, 444; number of HCCs, 30; number of composite endpoints, 47.

^b^Composite endpoint consists of development of HCC, decompensation, or mortality.

**Figure 2:**
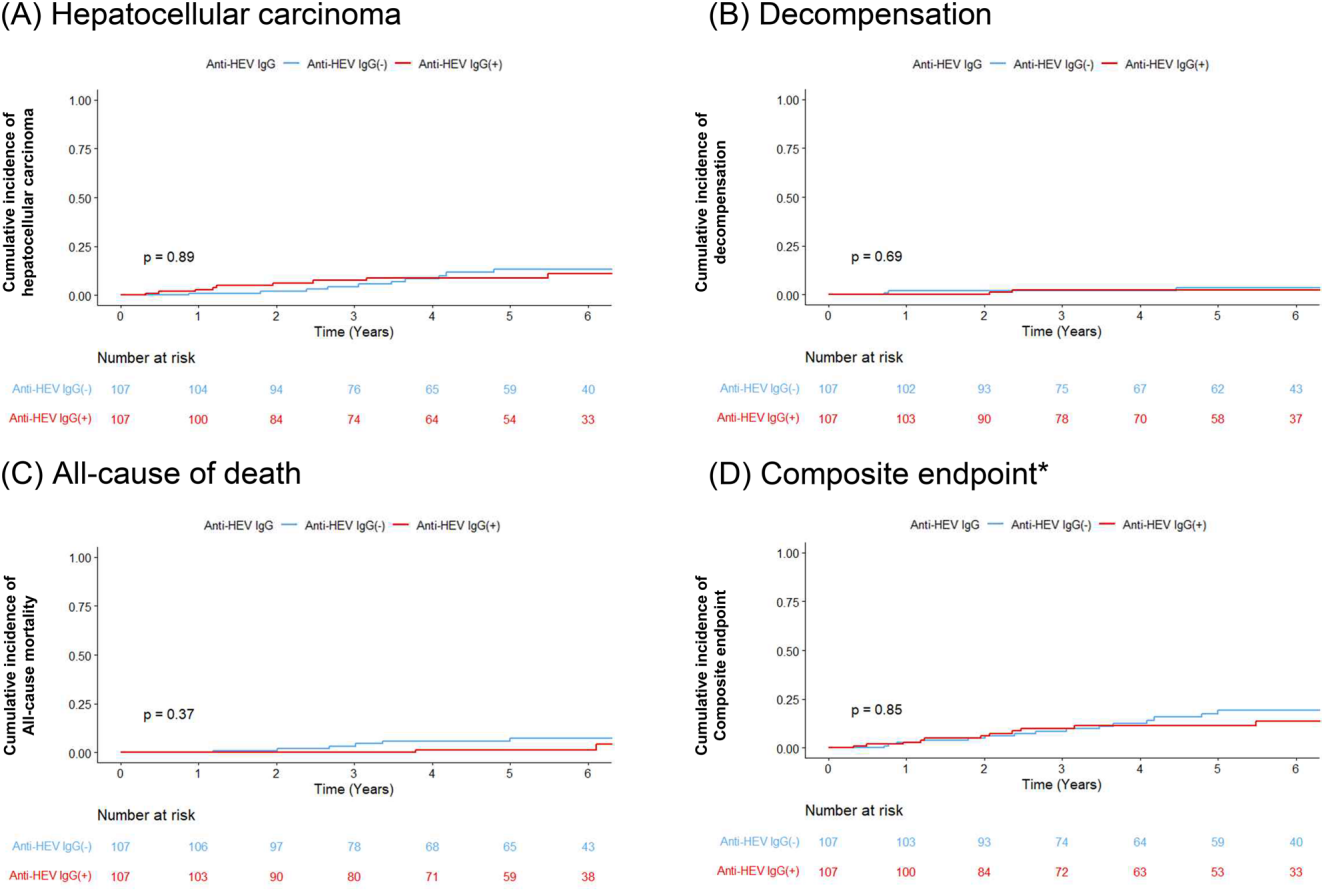
Cumulative incidence of hepatocellular carcinoma, decompensation, all-cause mortality, and composite endpoint in propensity score matched cohort with chronic hepatitis C infection. *Composite endpoint included hepatocellular carcinoma development, hepatic decompensation, and mortality.

After propensity score matching, 107 matched pairs were selected with no significant between-group differences in all baseline variables (Supplementary Table 1). In this propensity score matched cohort, there was no significant difference between the anti-HEV positive and negative groups in terms of HCC development, hepatic decompensation, or all-cause mortality (Fig. 2).

## Discussion

In this prospective study, we demonstrated a 33.3% prevalence of anti-HEV IgG in patients infected with chronic HCV. The independent factors associated with anti-HEV IgG positivity were patient age, male sex, and obesity, while the presence of liver cirrhosis was not associated with anti-HEV IgG positivity in either the multivariable analysis or propensity matching analysis. The incidence rate of anti-HEV IgG seroconversion was 0.98/100 person-years, and all seroconversions developed in patients that were aged at least 40. Anti-HEV IgG positivity at baseline or during follow-up (seroconversion) did not show a significantly adverse effect on HCC, hepatic decompensation, or mortality risks in this study population. The seroreversion rate was 1.2/100 person-years.

The anti-HEV IgG antibody prevalence among 2,450 patients aged 10 to 55 years from 2007 to 2009 in South Korea was reported to be 5.9% (2.4% in the 30s age group, 12.0% in the 40s age group, and 20.9% in the 50–55 years age group), similar to that of most low-endemic Asian-Pacific regions^9^. However, another study including 497 people aged 10–99 years in the National Health and Nutrition Examination Survey showed an anti-HEV IgG prevalence of 9.4% in 2005 (7.1% in 30s, 23.1% in 40–59 years, and 52.9% in over 60 years)^8^. Because there was no epidemic hepatitis E infection recently, there were only a few outdated prevalence data of anti-HEV IgG positivity in South Korea. Our study demonstrated updated data in Korean after 2013, even though we included only chronic hepatitis C patients in this study.

Considering that our study population’s mean age was 58.2 years and that the prevalence of anti-HEV increases with age, the anti-HEV prevalence in the chronic hepatitis C patients in this study did not appear to be higher than that in the general population of South Korea. This increasing trend of anti-HEV positivity according to age, especially in those > 40 years of age, has commonly been reported worldwide in those without^10,11^ and with chronic liver disease^12^. In individuals over the age of 60, the prevalence has been reported to be 21–56% in other Asian countries^13,14^, similar to our results (46.4%); however, the prevalence was higher than that in older Western populations (approximately 15%)^11,15,16^.

Because chronic liver disease affects the patient’s general health and immune status, those patients tend to be more susceptible to other hepatitis virus infections. Nonetheless, whether the anti-HEV prevalence in chronic liver disease patients is different among various etiologies is not clear. A previous study suggested that the anti-HEV IgG prevalence is higher in liver cirrhosis patients due to alcoholic (9.4%) or autoimmune liver disease (13.3%) compared with that in patients with hepatitis B viral disease (4.2%)^17^. According to the US data from the 2011–2018 National Health and Nutrition Examination Survey, the anti-HEV IgG seroprevalence of alcoholic liver disease patients (6.6%) was not significantly different from that of HCV-infected patients (8.7%), but HBV-infected patients showed a higher prevalence (19.9%)^17^. In contrast, several previous studies have documented that the prevalence of the anti-HEV antibody was higher in patients with chronic hepatitis C than in patients with chronic hepatitis B or in healthy controls^18,19^. Nonetheless, the results were age-unadjusted in those studies, and the patients in the chronic hepatitis C group were older patients in the other groups. Our data were analyzed after adjustment for subjects’ age and sex, but the present findings supported that anti-HEV antibody prevalence was not relatively higher in chronic hepatitis C patients than in the general population, unlike in chronic hepatitis B patients^17–19^. Therefore, the relationship between the cause of liver disease and anti-HEV prevalence is not clear; however, it is probably not significant.

There are also controversial reports on the prevalence of anti-HEV in patients with liver cirrhosis. A Chinese study showed that 6.49% of cirrhotic patients were positive for anti-HEV IgG, which is higher than the 1.33% positivity rate of chronic hepatitis patients^17^. Of cancer patients with underlying chronic liver disease, cirrhotic patients had an anti-HEV prevalence of 19%, which was higher than that in noncirrhotic patients (6%)^18^. Nonetheless, another study analyzing 1050 chronic hepatitis C patients with advanced fibrosis from the Hepatitis C Antiviral Long-Term Treatment Against Cirrhosis (HALT-C) trial did not show significant differences for model for end-stage liver disease scores (7.47 ± 1.50 vs. 7.31 ± 1.45, p = 0.42) or APRI (2.14 ± 1.63 vs. 2.11 ± 1.92, p = 0.92) between the anti-HEV positive and negative groups^7^. In the study, the only predictive factor for the presence of anti-HEV antibodies was age (p = 0.009). Similarly, our study found that the presence of a clinical diagnosis of liver cirrhosis or two kinds of noninvasive hepatic fibrosis markers did not predict anti-HEV positivity after adjusting for age. However, when we analyzed the subgroup under 60 years old, more patients with cirrhosis were positive for anti-HEV IgG (27.3%) than those without cirrhosis (16.4%, p = 0.037, data not shown). This finding suggested that cirrhotic conditions may confer more susceptibility to HEV infection^20^, but lifetime exposure is a much more decisive risk factor for HEV infection in chronic liver disease patients.

The present study documented the anti-HEV IgG seroconversion incidence rates for the first time in Korea. Only a few studies have been published about HEV antibody seroconversion rates, and all of them have analyzed the incidences in a healthy population. According to a previous study from China, the anti-HEV IgG seroconversion incidence was 17/1000 person-years^21^ in pregnant women. Of 1019 German blood donors, the seroconversion incidence of anti-HEV IgG was 0.35% per year^22^. Nonetheless, these studies were not comparable with our study because our study population was much older (58.2 ± 11.5 years old) than the those of the other studies (26.4 ± 4.1 years old in pregnant women^21^ and 41.5 (range 18—70) years old in blood donors^22^). The aforementioned HALT-C study^7^ (mean age 51 years, male 71%, 40% with liver cirrhosis) is the only study that reported a seroconversion rate in chronic hepatitis C patients of 2.5% per 5.1 years. Although direct comparison is impossible, the anti-HEV IgG seroconversion incidence in our patients (2.7% in 2.4 years, 0.98/100 person-years) seemed to be higher than that in the HALT-C study population. This may be related to the general prevalence of anti-HEV among the population and differences in eating behaviors.

In our data, HEV superinfection did not deteriorate the clinical courses of chronic hepatitis C patients. Despite evidence that symptomatic acute hepatitis E increases liver-related mortality in chronic liver disease patients^23^, the impact of subclinical HEV superinfection on chronic hepatitis C progression is currently unknown. In chronic hepatitis B patients, several studies^6,24^ have revealed that HEV coinfection can worsen clinical outcomes. According to a previous cohort study, 46 (2.2%) chronic hepatitis B patients developed HEV coinfection during the follow-up. Additionally, the HEV coinfected patients had an approximately 5 times higher risk of liver-related death after adjusting for age, sex, and HBV-related factors^6^. Unlike the findings in HBV patients, a large cohort study using the HALT-C trial data described similar results to those of our study; 21% of the patients tested positive for the anti-HEV antibody, showing that HEV coinfection did not predict hepatic decompensation (p = 0.70) during the 5.1 years of the follow-up period. A recent study documented that HEV replication was inhibited by HCV and vice versa using a coreplication in vitro model^25^. The authors also confirmed that the protease nonstructural protein 3/4A of HCV determines the interference inhibiting HEV replication. Taken together, we can assume that a preexisting HCV infection might attenuate the clinical severity of acute hepatitis E by viral interference and be protective from HEV-induced hepatic decompensation. However, acute severe hepatitis, regardless of the cause superimposed on decompensated cirrhosis, may increase the liver-related mortality of liver cirrhosis.

Seroreversion of anti-HEV IgG measured by Wantai ELISA was reported as 1.8% in 223 HCV mono- and HCV/HIV-coinfected patients in the US during a mean follow-up time of 24 months^26^. Another study showed a seroreversion rate of 8.8/1000 persons per year in the general population of the US^27^, while the seroreversion rate was 2.9/1000 persons per year in a non-HIV-infected cohort in Germany^28^. Compared to those Western studies, our results showed a higher seroreversion rate of 12/1000 persons-year, which may be related to HCV inference of HEV. Antibody avidity may be related to seroconversion or seroreversion, although limited studies have reported conflicting results^26^. Moreover, the reliability of HEV serology has been a major concern to directly compare the results from different populations. There is no gold standard test for HEV infection yet, and discrepancies between HEV-specific T-cell response and anti-HEV antibody reaction have been repeatedly reported^29,30^. Thus, anti-HEV IgG seroreversion and persistence of anti-HEV IgM for several years^31,32^ may complicate epidemiologic studies.

This study had several limitations. First, we selected patients whose serial samples were available at the 12-month interval. Thus, patients lost to follow-up or with poor compliance with blood sampling were excluded from the study population. Second, anti-HEV IgG is not a confirmatory test for HEV infection, but anti-HEV IgM, HEV RNA or genotype were not tested because none of them was suspected to have acute hepatitis at the time of blood sampling. All anti-HEV IgG positive subjects had normal liver enzyme levels in the samples. Nonetheless, a new occurrence of antibodies can be assumed to be a recent exposure to the HEV. Third, this study did not collect information about HEV risk factors, such as recent contact with animals and dietary or water supply history. Moreover, our questionnaire did not include some high-risk occupations for HEV infection, including animal-related occupations. Nevertheless, this information was not critical in our study because none of the detected acute hepatitis E cases needed an epidemiologic investigation.

In conclusion, approximately one-third of chronic HCV patients were coinfected with HEV, while the seroconversion and seroreversion rates were 0.98 and 1.22/100 person-years, respectively. HEV coinfection did not affect adverse hepatic outcomes or mortality, but future studies investigation the relationship between HEV and HCV are warranted.

## Methods

### Patients

**Figure 3:**
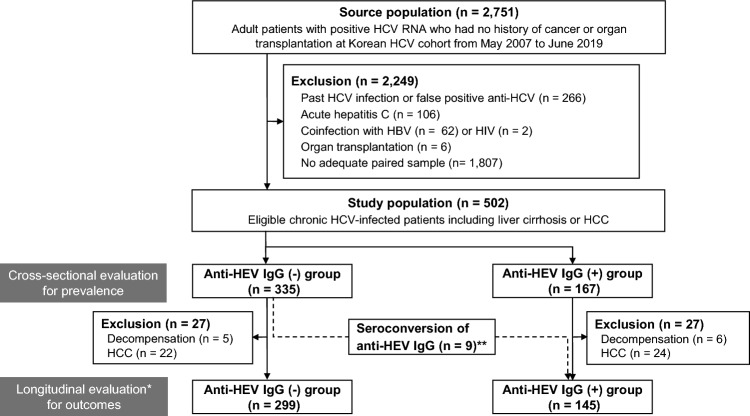
Patients flowgram. *Patients with decompensated liver cirrhosis (dcLC) and hepatocellular carcinoma (HCC) were excluded from outcome analysis. **One HCC patient showed anti-HEV IgG seroconversion. HCV, hepatitis C virus; RNA, ribonucleic acid; HBV, hepaitis B virus; HIV, human immunodeficiency virus; HCC, hepatocellular carcinoma; HEV, hepatitis E virus; IgG; immunoglobulin G.

A total of 502 viremic HCV-infected patients with serial blood samples collected at intervals of ≥ 12 months were selected from the Korea HCV cohort study subjects. This cohort was an established prospective, multicenter cohort funded by the Korean National Institute of Health since 2007^33^. Because serial blood sample collection began after 2013, this study included those who enrolled in the prospective cohort between Jul 2013 and Dec 2018 from 5 tertiary hospitals. The patient selection and overall outcomes according to anti-HEV positivity are summarized in Fig. 3. We excluded subjects with acute hepatitis C (n = 106), coinfection with hepatitis B virus (n = 62) or human immunosuppressive virus (n = 2), patients receiving immunosuppressive therapy after organ transplantation (n = 6), and without an adequate paired plasma sample (n = 1,807). For the cross-sectional study estimating anti-HEV IgG prevalence, 502 patients were included. However, for the longitudinal study on the outcome evaluation between anti-HEV positive and negative groups, the patients with decompensated cirrhosis (n = 11) or HCC (n = 46) at enrollment were excluded. Therefore, 444 patients were included in the outcome study (Fig. 3).

The study protocol was approved by the Institutional Review Board of Seoul National University Bundang Hospital (B-0706-046-002). The study was performed in accordance with the Declaration of Helsinki and all participants provided informed written consent of participation.

### Data collection and blood sampling

At enrollment, patients were interviewed by trained research coordinators using a standardized questionnaire which collected information including demographics, education, occupation, smoking status, alcohol intake, comorbid diseases, and lifetime exposure to risk factors for HCV infection. Laboratory and radiological data at enrollment and follow-up visits were retrieved from electronic medical records and entered into the established electronic case report form on the authorized website of the Korean Centers for Disease Control Korea HCV cohort study (http://is.cdc.go.kr) by the research coordinators in the five hospitals. In addition, independent researchers audited the data regularly to ensure quality control of multicenter data.

The patients were followed every 3–12 months by in-person or telephone visits. The follow-up laboratory and clinical outcome data were entered into the eCRFs. Additionally, indirect liver fibrosis markers such as aspartate aminotransferase (AST) to platelet ratio index (APRI)^34^ and fibrosis-4 index (FIB-4)^35^ were calculated at baseline and follow-up. Antiviral treatment was carried out at the physicians’ discretion and sustained virological response (SVR) was evaluated.

The sampling of a total blood volume of 16 ml for research use has been mandatory at enrollment since 2013; however, follow-up sampling was optional under the informed consent. Therefore, the sampling interval was not regular for each subject. After blood sampling, plasma was separated and securely transferred to the Seoul Central Laboratory within 24 h and kept in a deep freezer (− 70 °C) until usage.

### Anti-HEV IgG test

Anti-HEV IgG was measured by commercial enzyme-linked immunosorbent assay (ELISA, Wantai Biopharm, Beijing, China) according to the manufacturer’s instructions using the frozen paired plasma samples. If the absorbance value (optical density at 450 nm, OD_450_) of sample/cutoff (negative control + 0.16) was ≥ 1, it was considered as a positive result. All tests were duplicated, and the final anti-HEV IgG titer was the mean value of the duplicated tests. Follow-up measurements were performed in all subjects regardless of the baseline anti-HEV IgG result.

### Calculation of prevalence, seroconversion, and seroreversion incidence rates of HEV infection

Baseline anti-HEV IgG prevalence was calculated as the number of anti-HEV IgG-positive patients divided by the number of subjects in each group and indicated as a percentage. The incidence rate of HEV infection was defined as the number of patients with seroconversion (from anti-HEV IgG negative to positive) divided by the total follow-up period (person-years) among the patients whose baseline anti-HEV IgG was negative. The seroreversion rate was defined as the number of patients with seroreversion divided by the total follow-up period in person-years among the patients whose baseline anti-HEV IgG was positive. Incidence rates were indicated by the calculated 95% confidence interval (CI).

### Evaluation of liver-related outcomes

Outcomes were defined as the development of hepatic decompensation, HCC, and death/liver transplantation (LT). Hepatic decompensation was defined as complications of portal hypertension, including ascites, bleeding, or encephalopathy. HCC was diagnosed according to pathology or typical imaging criteria from the Korean Liver Cancer Association guidelines^12^. All-cause mortality or LT was documented by reviewing medical records and physician-confirmed death certificate data obtained from the Statistics Korea mortality database, which has the most reliable data on mortality.

To calculate the three clinical outcomes, patients with decompensated cirrhosis or HCC at baseline were excluded. The index date was defined as the date of baseline blood sampling, and the end of follow-up was defined as the date of the last follow-up or June 30, 2020.

### Statistical analysis

To compare the characteristics of anti-HEV IgG-negative and -positive groups, we used descriptive statistics, the χ^2^ test (for categorical variables), and Student’s t test (for continuous parametric variables). In addition, univariate and multivariate analyses using logistic regression were performed to identify factors related to anti-HEV positivity.

During the follow-up period, the anti-HEV IgG sero-conversion or -reversion incidence rates were compared between subgroups using an exact method based on the Poisson distribution.

To evaluate the long-term outcomes, patients with HCC and decompensated cirrhosis evaluated at baseline were excluded. To define the outcome of incident cases during follow-up, the index date was reset using a pseudo-Kaplan‒Meier method with a clock reset procedure. The data of seroreverted patients were censored at that time point. A time-varying Cox regression model was used to determine the factors associated with outcomes, and the adjusted hazard ratio was estimated for the entire cohort. In the models, baseline variables (sex, body mass index, alcohol, smoking, HCV genotype) were adjusted, and age, antiviral treatment, laboratory data, achievement of SVR, and seroconversion of anti-HEV IgG were considered time-dependent variables. To confirm the multivariate analysis results, we adjusted for significant differences in characteristics at the time of baseline anti-HEV IgG testing by propensity score (PS) matching for all possible variables. We used nearest-neighbor matching with a caliper size of 0.1 and matched the patients using a 1:1 ratio. Covariate balance was considered to be achieved if the absolute standardized difference between the two groups was ≤ 0.2.

All P values were two-sided, and a value of P < 0.05 was considered significant. Stata version 16.0 (College Station, TX, USA) and R (version 4.0.4; http://cran.r-project.org/) software were used for statistical analyses. The R package *MatchIt* was used for matching analyses.

### Supplementary Information

Supplementary Figures.

Supplementary Table S1.

## Data Availability

The datasets generated and analyzed during the current study are not publicly available due to the policy of Korea Disease Control and Prevention Agency. It can be available after the approval by the committee of National Institute of Infectious Disease, National Institute of Health with reasonable request submission. Please contact the corresponding author.

## Abbreviations

HCV: Hepatitis C virus
HEV: Hepatitis E virus
IgG: Immunoglobulin G
HCC: Hepatocellular carcinoma
AST: Aspartate aminotransferase
APRI: Aspartate aminotransferase to platelet ratio index
FIB-4: Fibrosis-4 index
SVR: Sustained virologic response
ELISA: Enzyme-linked immunosorbent assay
OD_450_: Optical density at 450 nm
CI: Confidence interval
LT: Liver transplantation
PS: Propensity score
DAA: Direct acting antivirals
PT INR: Prothrombin time international normalized ratio
OR: Odds ratio
HR: Hazard ratio
HALT-C: Hepatitis C antiviral long-term treatment against cirrhosis
