# Supplementary material for: Prevalence, incidence, and outcomes of hepatitis E virus coinfection in patients with chronic hepatitis C
Source: Sci Rep. 2023 Aug 21;13:13632. doi: 10.1038/s41598-023-39019-3 (PMC10442446; doi:10.1038/s41598-023-39019-3)
Supplement: Supplementary file 1 — Supplementary Figures. [file 41598_2023_39019_MOESM1_ESM.pptx]

## Slide 1
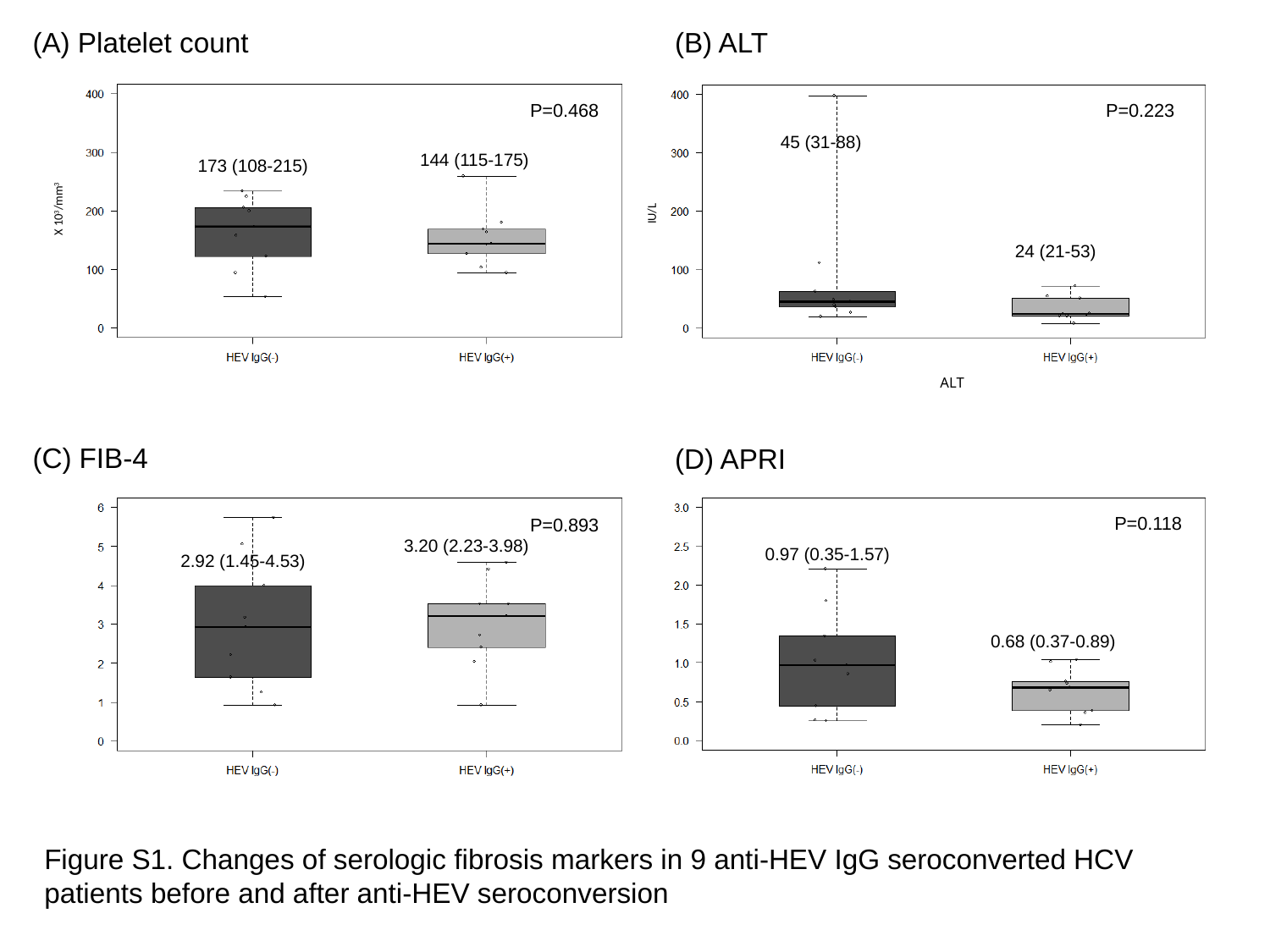

(A) Platelet count
(B) ALT
X 103/mm3
P=0.468
P=0.223
IU/L
45 (31-88)
144 (115-175)
173 (108-215)
24 (21-53)
ALT
(C) FIB-4
(D) APRI
P=0.118
P=0.893
3.20 (2.23-3.98)
0.97 (0.35-1.57)
2.92 (1.45-4.53)
0.68 (0.37-0.89)
Figure S1. Changes of serologic fibrosis markers in 9 anti-HEV IgG seroconverted HCV patients before and after anti-HEV seroconversion

## Slide 2
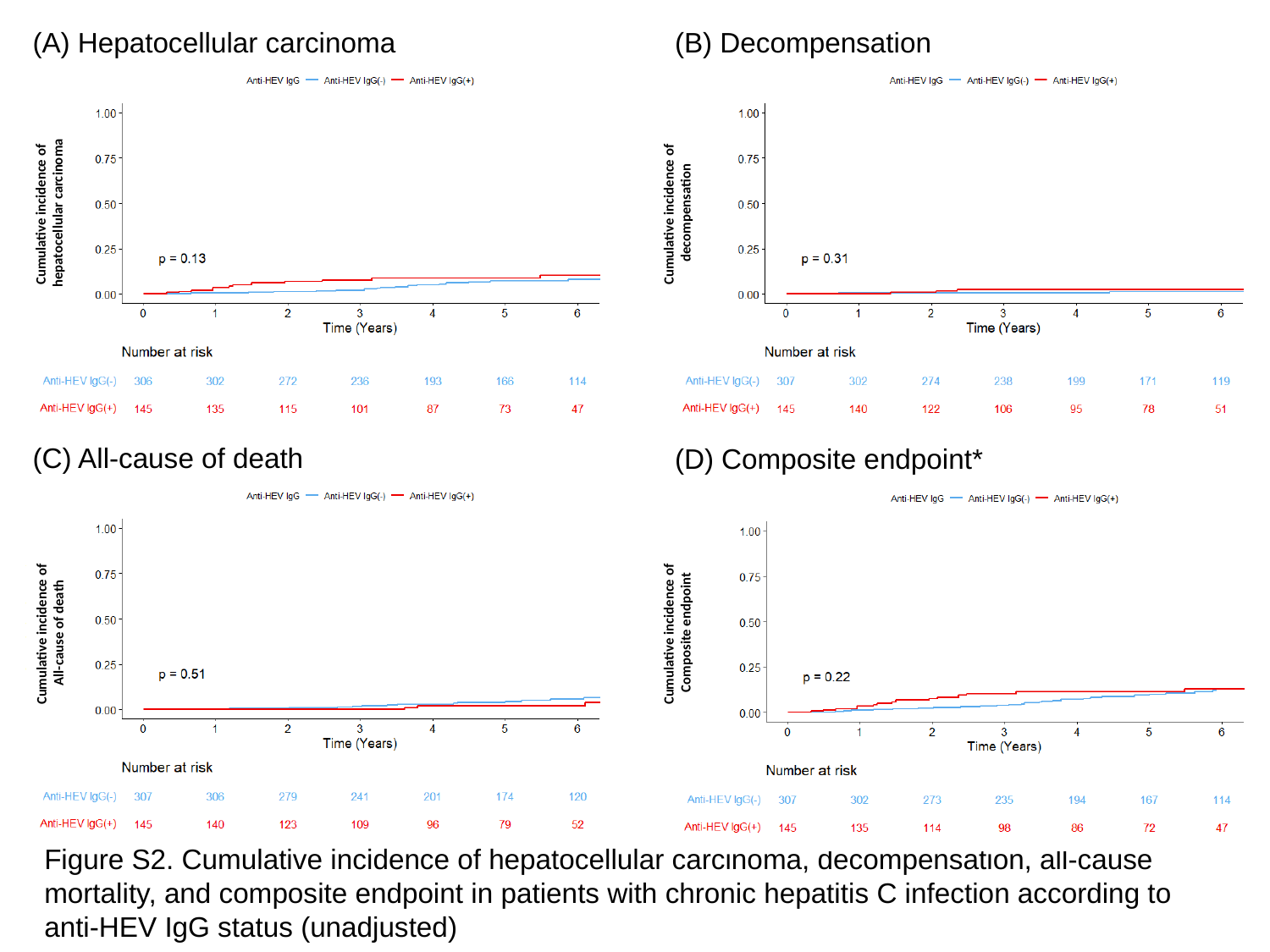

(A) Hepatocellular carcinoma
(B) Decompensation
Cumulative incidence of
hepatocellular carcinoma
Cumulative incidence of
decompensation
(C) All-cause of death
(D) Composite endpoint*
Cumulative incidence of
All-cause of death
Cumulative incidence of
Composite endpoint
Figure S2. Cumulative incidence of hepatocellular carcinoma, decompensation, all-cause mortality, and composite endpoint in patients with chronic hepatitis C infection according to anti-HEV IgG status (unadjusted)
