# Supplementary material for: Prevalence, incidence, and outcomes of hepatitis E virus coinfection in patients with chronic hepatitis C
Source: Sci Rep. 2023 Aug 21;13:13632. doi: 10.1038/s41598-023-39019-3 (PMC10442446; doi:10.1038/s41598-023-39019-3)
Supplement: Supplementary file 2 — Supplementary Table S1. [file 41598_2023_39019_MOESM2_ESM.docx]

**Supplementary Table S1. Characteristics of chronic hepatitis and compensated cirrhosis**

| **Characteristics** | **Before adjustment** | | | **Propensity-score matched** | | |
| --- | --- | --- | --- | --- | --- | --- |
|  | **Anti-HEV IgG (-)**  **(n=299)** | **Anti-HEV IgG (+)**  **(n=145)** | **SMD** | **Anti-HEV IgG (-)**  **(n=107)** | **Anti-HEV IgG (+)**  **(n=107)** | **SMD** |
| Age | 54.8 ± 11.5 | 63.2 ± 8.9 | 0.949 | 61.1 ± 10.0 | 61.4 ± 8.9 | 0.040 |
| Male | 128 (42.8) | 81 (44.1) | 0.262 | 51 (47.7) | 51 (47.7) | 0.000 |
| Liver cirrhosis | 81 (27.1) | 58 (40.0) | 0.264 | 41 (38.3) | 36 (33.6) | 0.095 |
| APRI | 0.54 (0.33 – 1.23) | 0.70 (0.41 – 1.29) | 0.090 | 0.79 (0.44 – 1.43) | 0.70 (0.40 – 1.23) | 0.154 |
| FIB-4 | 2.02 (1.31 – 3.94) | 2.87 (1.37 – 4.35) | 0.238 | 3.11 (1.85 – 1.43) | 2.80 (1.90 – 4.11) | 0.121 |
| HCV genotype |  |  | 0.161 |  |  | 0.038 |
| 1 | 158 (52.8) | 65 (44.8) |  | 53 (49.5) | 51 (47.7) |  |
| 2 | 130 (43.5) | 74 (51.) |  | 52 (48.6) | 53 (49.5) |  |
| Others or missing | 11 (3.7) | 6 (4.1) |  | 2 (1.9) | 3 (2.8) |  |
| HCV RNA, Log_10_ IU/mL | 5.80 (4.37 – 6.44) | 5.54 (3.52 – 6.38) | 0.146 | 5.91 (4.81 – 6.44) | 5.37 (4.24 – 6.40) | 0.162 |
| Treatment history with SVR | 43 (14.4) | 25 (17.3) | 0.067 | 12 (11.2) | 19 (15.9) | 0.101 |
| Diabetes mellitus | 42 (14.0) | 24 (16.6) | 0.067 | 17 (15.9) | 20 (18.7) | 0.075 |
| Body mass index, kg/m^2^ | 23.7 ± 3.3 | 24.3 ± 3.4 | 0.171 | 24.4 ± 3.9 | 24.5 ± 3.2 | 0.002 |
| Heavy alcohol consumption | 63 (21.1) | 24 (16.6) | 0.122 | 26 (24.3) | 19 (17.8) | 0.176 |
| Ever Smoking | 124 (41.5) | 62 (42.8) | 0.026 | 50 (46.7) | 46 (43.0) | 0.076 |
| Laboratory findings |  |  |  |  |  |  |
| WBC, ×1,000/mm^3^ | 5.2 (4.3 – 6.4) | 5.1 (4.1 – 6.5) | 0.063 | 5.4 (4.5 – 6.7) | 5.3 (4.4 – 6.5) | 0.079 |
| Hemoglobin, g/dL | 13.8 (12.8 – 14.9) | 13.7 (12.4 – 14.7) | 0.151 | 13.7 (12.8 – 14.8) | 13.7 (12.5 – 14.8) | 0.043 |
| Platelet, ×1,000/mm^3^ | 179 (139 - 223) | 163 (123 – 207) | 0.316 | 166 (127 – 209) | 169 (129 – 209) | 0.083 |
| Albumin, g/dL | 4.3 (4.0 – 4.5) | 4.2 (3.9 – 4.5) | 0.193 | 4.2 (3.9 – 4.4) | 4.2 (4.0 – 4.5) | 0.164 |
| Bilirubin, mg/dL | 0.7 (0.6 – 1.0) | 0.8 (0.6 – 1.0) | 0.037 | 0.8 (0.6 – 1.0) | 0.7 (0.5 – 1.0) | 0.099 |
| AST, IU/L | 41 (28 – 65) | 41 (29 – 70) | 0.040 | 48 (32 – 76) | 45 (30 – 70) | 0.250 |
| ALT, IU/L | 35 (23 – 58) | 32 (21 – 59) | 0.108 | 38 (22 – 61) | 34 (21 – 63) | 0.184 |
| Prothrombin Time, INR | 1.04 (0.99 – 1.11) | 1.04 (0.99 – 1.11) | 0.189 | 1.09 (1.03 – 1.15) | 1.06 (1.00 – 1.10) | 0.199 |
| Creatinine, mg/dL | 0.80 (0.67 – 0.97) | 0.90 (0.70 – 1.00) | 0.077 | 0.82 (0.67 – 1.00) | 0.89 (0.70 – 1.00) | 0.019 |
| Alpha-fetoprotein, ng/dL | 3.5 (3.2 – 6.8) | 3.3 (2.1 – 6.6) | 0.031 | 3.8 (2.4 – 8.7) | 3.2 (2.04 – 6.8) | 0.055 |
| Treatment during follow-up |  |  | 0.051 |  |  | 0.039 |
| IFN-based, no SVR | 5 (1.7) | 1 (0.7) |  | 1 (0.9) | 1 (0.9) |  |
| IFN-based, SVR | 22 (7.4) | 12 (8.3) |  | 4 (3.7) | 8 (7.5) |  |
| DAA, no SVR | 10 (3.3) | 5 (3.4) |  | 3 (2.8) | 5 (4.7) |  |
| DAA, SVR | 175 (58.5) | 82 (56.6) |  | 64 (59.8) | 62 (57.9) |  |
| Follow-up period, year | 5.4 (3.2 – 6.8) | 5.4 (3.0 – 6.8) |  | 5.5 (2.9 - 6.8) | 5.5 (3.0 - 6.8) |  |

HEV, hepatitis E virus; IgG, immunoglobulin G; APRI, aspartate aminotransferase platelet ratio index; HCV, hepatitis C virus; RNA, ribonucleic acid; SVR, sustained virologic response; WBC, white blood cell; AST, Aspartate aminotransferase; ALT, Alanine aminotransferase; IFN, interferon; DAA, direct acting antivirals
